# Polysymptomatology in Pediatric Patients Receiving Palliative Care Based on Parent-Reported Data

**DOI:** 10.1001/jamanetworkopen.2021.19730

**Published:** 2021-08-05

**Authors:** Chris Feudtner, Russell Nye, Douglas L. Hill, Matt Hall, Pam Hinds, Emily E. Johnston, Sarah Friebert, Ross Hays, Tammy I. Kang, Joanne Wolfe

**Affiliations:** 1 Justin Ingerman Center for Palliative Care, The Children’s Hospital of Philadelphia, Philadelphia, Pennsylvania; 2 Department of Pediatrics, Medical Ethics and Health Policy, The Perelman School of Medicine at the University of Pennsylvania, Philadelphia; 3 Children’s Hospital Association, Lenexa, Kansas; 4 Department of Nursing Science, Professional Practice & Quality, Children’s National Hospital, Washington, DC; 5 Department of Pediatrics, George Washington University, Washington, DC; 6 Division of Hematology and Oncology, Department of Pediatrics, University of Alabama at Birmingham, Birmingham; 7 Division of Palliative Care, Department of Pediatrics, Akron Children’s Hospital and Rebecca D. Considine Research Institute, Akron, Ohio; 8 Division of Bioethics and Palliative Care, Department of Pediatrics, University of Washington School of Medicine, Seattle; 9 Department of Pediatrics, Section of Palliative Care, Texas Children’s Hospital and Baylor College of Medicine, Houston; 10 Department of Psychosocial Oncology and Palliative Care, Dana Farber Cancer Institute, Boston, Massachusetts; 11 Department of Pediatrics, Boston Children’s Hospital, Boston, Massachusetts

## Abstract

**Importance:**

Pediatric palliative care treats patients with a wide variety of advanced illness conditions, often with substantial levels of pain and other symptoms. Clinical and research advancements regarding symptom management for these patients are hampered by the scarcity of data on symptoms as well as an overreliance on clinician report.

**Objective:**

To provide a detailed description of the symptoms among patients receiving pediatric palliative care based on parental report via a validated, structured symptom assessment measure.

**Design, Setting, and Participants:**

Baseline data for this cross-sectional analysis were collected between April 10, 2017, and February 5, 2020, from pediatric palliative care programs in 7 children’s hospitals located in Akron, Ohio; Boston, Massachusetts; Birmingham, Alabama; Houston, Texas; Minneapolis, Minnesota; Philadelphia, Pennsylvania; and Seattle, Washington. Data were collected in the hospital, outpatient, and home setting from patients 30 years of age or younger who were receiving pediatric palliative care at 1 of the study sites.

**Exposures:**

Analyses were stratified by patients’ demographic characteristics, including age, and by whether the patients had received a diagnosis of any of 10 non–mutually exclusive complex chronic condition categories.

**Main Outcomes and Measures:**

Twenty symptoms measured via the modified Memorial Symptom Assessment Scale, which scores the frequency and severity of any symptom that is present and provides a total symptom score.

**Results:**

Among the first 501 patients enrolled, the median age was 4.1 years (interquartile range, 0.8-12.9 years), 267 (53.3%) were male, and 356 (71.1%) were White. The most prevalent complex chronic conditions included gastrointestinal (357 [71.3%]), neurologic (289 [57.7%]), and cardiovascular (310 [61.9%]) conditions; 438 patients (87.4%) were technology dependent. Parents reported a mean (SD) of 6.7 (3.4) symptoms per patient and a median of 7 symptoms (interquartile range, 4-9 symptoms). A total of 367 patients (73.3%) had 5 or more symptoms. The 5 most prevalent symptoms were pain (319 [63.7%]; 95% CI, 59.4%-67.8%), lack of energy (295 [58.9%]; 95% CI, 54.5%-63.1%), irritability (280 [55.9%]; 95% CI, 51.5%-60.2%), drowsiness (247 [49.3%]; 95% CI, 44.9%-53.7%), and shortness of breath (232 [46.3%]; 95% CI, 41.9%-50.7%). Although older patients were reported by parents as having experienced more symptoms and having higher total symptom scores, variation across condition categories was relatively minor. Patients in the upper 10th percentile of total symptom scores had a median of 12.0 symptoms (interquartile range, 11-13).

**Conclusions and Relevance:**

In this cross-sectional study, most children receiving palliative care were experiencing polysymptomatology. An important subgroup of patients frequently experienced numerous severe symptoms. Assessment and management of patients with polysymptomatology are critical aspects of pediatric palliative care.

## Key Points

**Question:**

What symptoms do pediatric patients receiving palliative care experience, and how frequent and severe are the symptoms, according to parent report?

**Findings:**

In this cross-sectional analysis of 501 pediatric patients receiving palliative care, at baseline, parents reported a mean (SD) of 6.7 (3.4) symptoms per patient; although older patients had more symptoms and higher symptom scores, variation across disease categories was relatively minor. Patients in the upper 10th percentile of symptom frequency and severity scores had a median of 12 symptoms.

**Meaning:**

This study suggests that assessment and management of polysymptomatology is a critical aspect of pediatric palliative care.

## Teaser

This cross-sectional analysis describes symptoms among patients receiving pediatric palliative care based on parental report via a validated, structured symptom assessment measure.

## Introduction

Symptom assessment and management are foundational aspects of pediatric palliative care (PPC).^1^ Pediatric palliative care serves patients with a wide variety of severe illnesses and complex chronic conditions (CCCs) from birth to young adulthood and works to deliver care that addresses patients’ health care needs and aligns with patient and family goals.^2,3^ For children receiving home-based PPC services, parents’ top priority was the physical care of the child, including symptom management.^4^ As parents and PPC clinicians can attest, children receiving PPC experience an array of symptoms, often concurrently, and uncontrolled symptoms undermine both comfort and quality of life for children and their parents.^5^

Among patients receiving PPC, many basic but important questions regarding symptoms—incidence, prevalence, severity, and co-occurrence—have either no empirically based answers or answers limited to a particular group of patients or to physician-reported assessments.^6^ What is clear, given the existing evidence, is that, among children with serious illness, significant symptoms are common, often to a degree that is distressing to the patients and their caregivers. Among children with advanced cancer, parents and self-reporting patients often report substantial pain, fatigue, drowsiness, and irritability.^7,8^ Parents of children with serious central nervous system impairment report that their children experienced a mean of 3.2 symptoms, and at least half the children experienced pain or irritability, sleep problems, and gastrointestinal symptoms such as nausea and vomiting.^9^ Pediatric palliative care physicians report that substantial proportions of their patients experience cognitive impairment, speech impairment, sleep and fatigue problems, irritability, and pain.^10^

In this study, we sought to characterize the point prevalence, frequency, and severity of specific symptoms and the co-occurrence of symptom, among a large, diverse sample of patients receiving PPC. To do so, we analyzed baseline data from 501 patients enrolled in an ongoing cohort study, using a structured parent-report symptom assessment tool of 20 symptoms. Given the widely held beliefs among clinicians that symptoms among patients receiving PPC vary depending on underlying conditions and by age, we examined whether symptom metrics differed across patients with different CCCs and across age groups.

## Methods

### Study Design, Setting, and Participants

This cross-sectional study used parent-reported baseline data from the ongoing Pediatric Palliative Care Research Network’s Shared Data and Research (SHARE) 2-year prospective cohort study being conducted at 7 children’s hospitals across the United States. Patients were eligible if they were receiving PPC services and were younger than 30 years. Parents were eligible to participate if they were responsible for the patient, at least 18 years of age or a minor permitted by state law to consent for their own participation, and able to speak and understand English or Spanish. Institutional review boards at The Children’s Hospital of Philadelphia Research Institute, Boston Children’s Hospital, Seattle Children’s Research Institute, and Children’s Minnesota approved the conduct of this study. Parents and competent patients 18 years or older provided written informed consent. This study followed the Strengthening the Reporting of Observational Studies in Epidemiology (STROBE) reporting guidelines.

Enrollment began April 10, 2017, and ended December 18, 2020. The current sample represents the first 501 patients who enrolled and had evaluable baseline data collected by February 5, 2020. Of the 641 eligible patients and parents approached regarding the study, 501 (78.2%) agreed to participate and completed at least background information (including race and ethnicity, self-identified by participants, to assess for possible disparities in PPC^11,12,13,14^) and the symptom assessment measure from the baseline instruments.

### Data Collection

At study entry, parents completed a set of instruments either in a clinical care setting or at home, with data recorded on either paper or internet-based forms. Paper forms were subsequently double entered by study staff into the internet-based forms.

### Symptom Assessment Measure

Parent report of the child’s symptoms during the prior 7 days was measured using the previously validated Memorial Symptom Assessment Scale (MSAS) adapted for children^15^ and further adapted (PediQUEST [PQ]-MSAS) for children with serious illness.^7,16^ The measure exists in 2 versions; the PQ-MSAS for parents with children younger than 2 years assesses 20 different symptoms, and the PQ-MSAS for parents with children 2 years of age or older assesses 28 different symptoms. To compare how the 2 versions assessed these symptoms, see the eTable in Supplement 1. To have uniformity of symptoms assessed across study participants’ age groups, only the 20 symptoms assessed by both instruments were analyzed. The PQ-MSAS asks the reporter to rate, for each symptom, the presence of the symptom (yes or no), the frequency of the symptom (where 0 indicates never; 1, almost never; 2, sometimes; 3, a lot; and 4, almost always), and the severity of the symptom (where 0 indicates none; 1, slight; 2, moderate; 3, severe; and 4, very severe). Frequency and severity were transformed into scores ranging from 0 to 100 by multiplying the raw score by 25. Symptom scores for each symptom were calculated by adapting and modifying methods previously used^7,16,17^ to account for assessment differences of specific symptoms and between the age groups. Specifically, a patient’s symptom score for each symptom was calculated as the mean of frequency and severity values for the symptoms in which both these attributes were assessed, as the severity score for the 3 symptoms for which only severity was uniformly assessed, and as the overall mean symptom score value among symptomatic patients for the 2 symptoms for which neither severity nor frequency was assessed (seizures and bleeding in the older age group). An individual’s total symptom score was the mean of all of the separate transformed symptom scores and could range from 0 to 100.

### CCC Designation

Enrolled patients’ medical record numbers were used to match study participants with their data in the Pediatric Hospital Information System (PHIS; Children’s Hospital Association) administrative database. Of the 501 participants, 472 had matching Pediatric Hospital Information System data, which included diagnoses recorded as *International Statistical Classification of Diseases and Related Health Problems, Tenth Revision, Clinical Modification* codes for any hospitalizations that occurred during the year prior to the date of baseline assessment completion and which were used to identify whether participating patients were included in any of the 12 different non–mutually exclusive CCC categories.^18^ For patients with no Pediatric Hospital Information System data, CCC categories were identified via medical record review.

### Statistical Analysis

Missing data in the variables used in this analysis were minimal (1 of 501 [0.2%]), except for time receiving PPC prior to enrollment (21 of 501 [4.2%]). Descriptive statistics were calculated to summarize the demographic and clinical characteristics of patients receiving PPC, including parent-reported patient symptoms. Differences in the number of symptoms and in the total symptom score by demographic and clinical characteristics were assessed using 2-tailed *t* tests, analysis of variance, and linear regression. Scatterplots, box and whisker plots, bar charts, and radar plots were generated to display symptom point prevalence, frequency, and severity across the sample and by age group and CCC category. An ordinary least-squares regression model was used to assess patients’ total symptom scores as a function of their symptom counts and age groups; for these analyses, α was set at .05. All statistical analyses were performed using Stata, version 16.0 (StataCorp).

## Results

Among the first 501 patients receiving PPC enrolled in SHARE (Table), the median age was 4.1 years (interquartile range, 0.8-12.9 years). Patients were primarily male (267 [53.3%]) and were identified by their parents as White (356 [71.1%]), Black (42 [8.4%]), Asian (15 [3.0%]), Native population (American Indian, Native Hawaiian, and/or Alaska Native; 25 [5.0%]), or other/multiple race (63 [12.6%]); 75 patients (15.0%) were identified as Hispanic. Patients’ health insurance coverage was government issued (205 [40.9%]), private (154 [30.7%]), or both government and private (131 [26.2%]); 5 parents (1.0%) reported no insurance, and 6 (1.2%) either chose not to disclose or had an unspecified other form of insurance.

**Table.** Patient Characteristics and Symptoms

Characteristic	No. (%)	No. of symptoms		Total symptom score	
		Mean (SD)	*P* value	Mean (SD)	*P* value
Entire sample	501 (100)	6.7 (3.4)		19.6 (11.7)	
Age^a^					
<1 mo	19 (3.8)	4.5 (3.2)	.001	12.3 (9.5)	.001
1-11 mo	121 (24.2)	6.3 (3.2)		17.8 (11.0)	
1-9 y	183 (36.5)	6.6 (3.4)		19.1 (11.8)	
10-17 y	147 (29.3)	7.1 (3.4)		21.8 (11.4)	
18-28 y	31 (6.2)	8.2 (3.7)		24.5 (12.8)	
Sex^a^					
Female	233 (46.5)	6.4 (3.5)	.11	18.8 (11.7)	.11
Male	267 (53.3)	6.9 (3.3)		20.4 (11.6)	
Race^a^					
White	356 (71.1)	6.6 (3.3)	.62	19.1 (11.3)	.51
Black	42 (8.4)	7.4 (3.3)		21.5 (11.5)	
Asian	15 (3.0)	6.7 (3.6)		20.3 (13.8)	
Native population	25 (5.0)	6.7 (4.6)		21.0 (14.7)	
Other^b^	63 (12.6)	7.0 (3.4)		21.4 (12.3)	
Ethnicity^a^					
Hispanic	75 (15.0)	6.1 (3.2)	.24	17.7 (11.2)	.28
Non-Hispanic	412 (82.4)	6.8 (3.4)		20.0 (11.8)	
Not indicated	13 (2.6)	6.5 (3.5)		18.6 (10.9)	
Technology dependent^c^	438 (87.4)	6.8 (3.4)	.02	20.2 (11.8)	.009
CCC type^c^					
Gastrointestinal	357 (71.3)	6.8 (3.4)	.32	20.2 (11.6)	.11
Neuromuscular	289 (57.7)	6.7 (3.6)	.45	20.4 (12.4)	.10
Cardiovascular	310 (61.9)	6.7 (3.5)	.91	19.7 (12.2)	.94
Metabolic	256 (51.1)	7.1 (3.3)	.001	21.3 (11.5)	.001
Respiratory	206 (41.1)	6.7 (3.4)	.98	19.9 (11.7)	.68
Renal	206 (41.1)	7.0 (3.4)	.14	20.6 (11.8)	.12
Congenital	209 (41.7)	7.1 (3.4)	.14	21.0 (12.1)	.03
Malignant neoplasm	150 (29.9)	6.9 (3.3)	.39	20.0 (11.2)	.65
Hematologic	175 (34.9)	6.8 (3.2)	.71	20.0 (11.2)	.61
Neonatal	117 (23.4)	6.6 (3.5)	.57	19.3 (11.6)	.75
Transplant	70 (14.0)	6.7 (3.6)	.69	18.6 (11.9)	.41
No. of CCCs^d^					
1	15 (3.0)	4.1 (2.6)	.02	10.9 (7.3)	.002
2	24 (4.8)	6.6 (3.6)		17.8 (12.0)	
3	39 (7.8)	6.2 (3.4)		17.0 (10.1)	
4	59 (11.8)	6.5 (3.4)		19.2 (12.0)	
5	105 (21.0)	6.8 (3.4)		20.5 (12.0)	
6	93 (18.6)	7.2 (3.4)		20.9 (11.9)	
7	83 (16.6)	6.7 (2.9)		19.4 (10.4)	
8	52 (10.4)	6.2 (3.7)		18.1 (11.9)	
9	23 (4.6)	7.3 (3.1)		23.7 (11.8)	
10	8 (1.6)	9.9 (3.5)		32.5 (13.2)	

Abbreviation: CCCs, complex chronic conditions.

^a^ Analysis of variance used to assess differences in means for the categorical variables.

^b^ Indicated as other, preferred not to answer, or multiple race.

^c^ The *t* test was used to assess differences of means for patients with each specific CCC type of diagnoses that differed from the mean of all other patients without that specific CCC type of diagnoses.

^d^ Ordinary least-squares linear regression was used to test whether the mean values increased as the number of CCCs increased.

Patients had been receiving PPC services on average for 1 year (mean, 363 days; range, 1 day to 14.6 years; eFigure in Supplement 1). Most patients (438 [87.4%]) were technology dependent (Table), and all patients had CCC diagnoses. The most prevalent (nonmutually exclusive) CCC categories were gastrointestinal (357 [71.3%]), cardiovascular (310 [61.9%]), and neurologic (289 [57.7%]). Patients had a median of 6 different CCC categories of diagnoses (interquartile range [IQR], 4-7 CCC categories).

**Figure 1.**
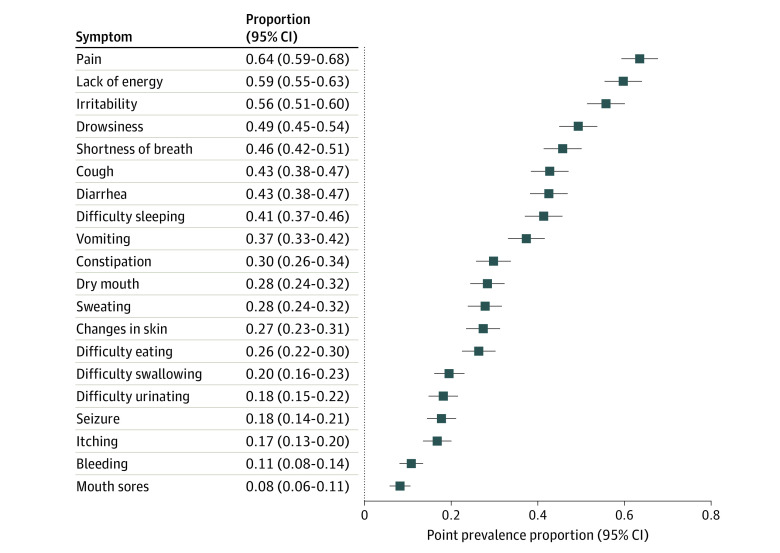
Point Prevalence of 20 Symptoms Based on Parent Report Horizontal lines indicate 95% CIs.

Among the 501 patients, according to parent report at the time of study entry, 488 (97.4%) were experiencing at least 1 of the 20 assessed symptoms. The point prevalences of these specific symptoms ranged broadly (Figure 1), with the 5 most prevalent symptoms being pain (319 [63.7%]; 95% CI, 59.4%-67.8%), lack of energy (295 [58.9%]; 95% CI, 54.5%-63.1%), irritability (280 [55.9%]; 95% CI, 51.5%-60.2%), drowsiness (247 [49.3%]; 95% CI, 44.9%-53.7%), and shortness of breath (232 [46.3%]; 95% CI, 41.9%-50.7%).

**Figure 2.**
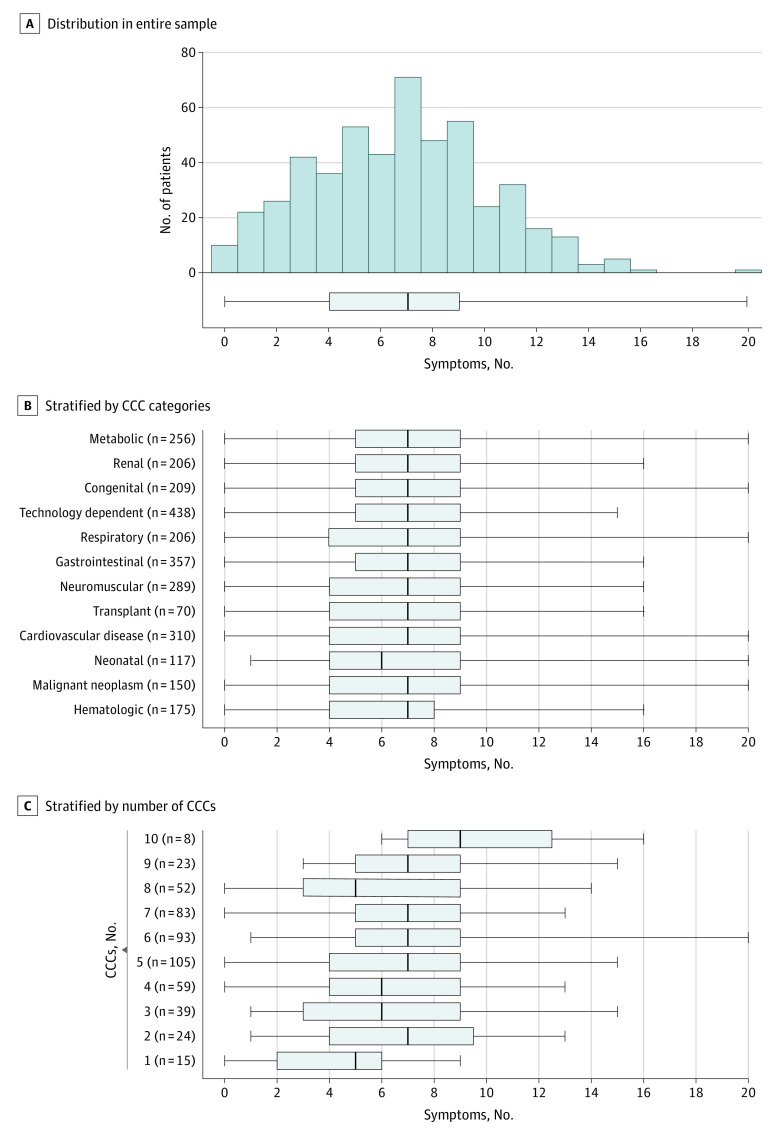
Frequency of Patients With Different Numbers of Reported Symptoms CCC indicates complex chronic conditions. Gray boxes indicate the interquartile range, vertical lines in the boxes indicate the median, and the whiskers indicate the range.

As reported by parents, patients were experiencing a mean (SD) of 6.7 (3.4) of the 20 symptoms (Table). A total of 367 patients (73.3%) had 5 or more symptoms. The number of symptoms increased steadily with increasing patient age and with regard to the amount of time receiving PPC services prior to enrollment; no associations were observed regarding sex, race, or ethnicity categories. Differences in the distribution of the number of different symptoms by patient clinical characteristics can be examined graphically in Figure 2. Figure 2A shows the overall distribution of symptoms, plotted as a histogram and as a box plot, with a median of 7 symptoms (IQR, 4-9 symptoms). Figure 2B shows the same box plot, stratified by patients who had specific categories of CCCs, with the number of patients in each strata annotated at the end of each upper range. Across all the CCC categoriess the median symptom counts (range, 6-7) and IQRs (25th percentile range, 4-5; 75th percentile range, 8-9) were similar. Figure 2C shows the same information stratified by groups of patients who had increasing numbers of different CCC categories, from 1 to 10 different CCC categories. Across these strata, while the median symptom counts and IQRs varied more than was seen across CCC categories, the number of symptoms increased only slightly (and not consistently) among patients with increasing numbers of CCC categories.

Regarding patients’ total symptom scores (the mean of symptom scores, rescaled on a 0-100 range) across the full sample, these scores ranged from 0 to 63.8 with a mean (SD) of 19.6 (11.7). Differences in total symptom scores by patient characteristics are also reported in the Table. These scores increased steadily with increasing patient age; no associations were observed regarding sex, race, or ethnicity categories, nor regarding time receiving PPC services (eFigure in Supplement 1). Although the mean (SD) scores were slightly higher than average for patients who were technology dependent and were highest among patients who had metabolic CCCs (21.3 [11.5]) and lower among patients with transplant CCCs (18.6 [11.9]), the mean scores ranged by only 3 points across all of the CCC categories.

**Figure 3.**
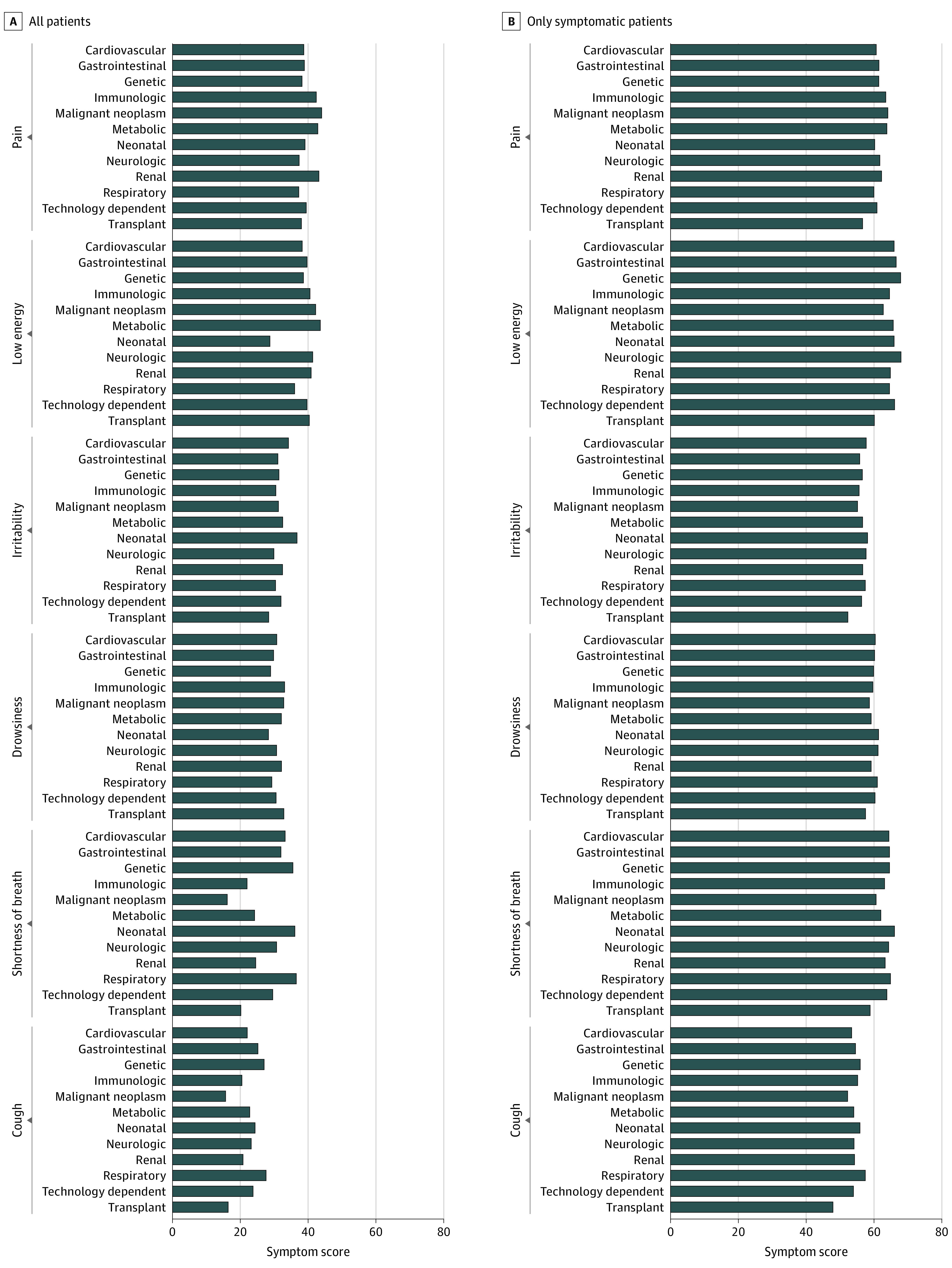
Symptom Scores Across Categories of Complex Chronic Conditions, for All Patients and Only Symptomatic Patients

We also examined the symptom scores for each symptom across patients with each of the CCC category diagnoses, for all patients (both asymptomatic and symptomatic) and for just patients who were experiencing the symptom (symptomatic). Figure 3 provides a bar graph for the 6 most commonly reported symptoms. Although the symptom scores differed slightly for all patients across the CCC categories, these differences were mostly due to different symptom prevalences in each CCC category. Examining the symptom scores among only the patients who were symptomatic, the mean symptom score for each symptom was essentially the same across CCC categories. For example, focusing on shortness of breath, among patients with malignant neoplasm CCCs, the prevalence was notably less, but among patients who were symptomatic, the intensity was the same as other CCC categories.

**Figure 4.**
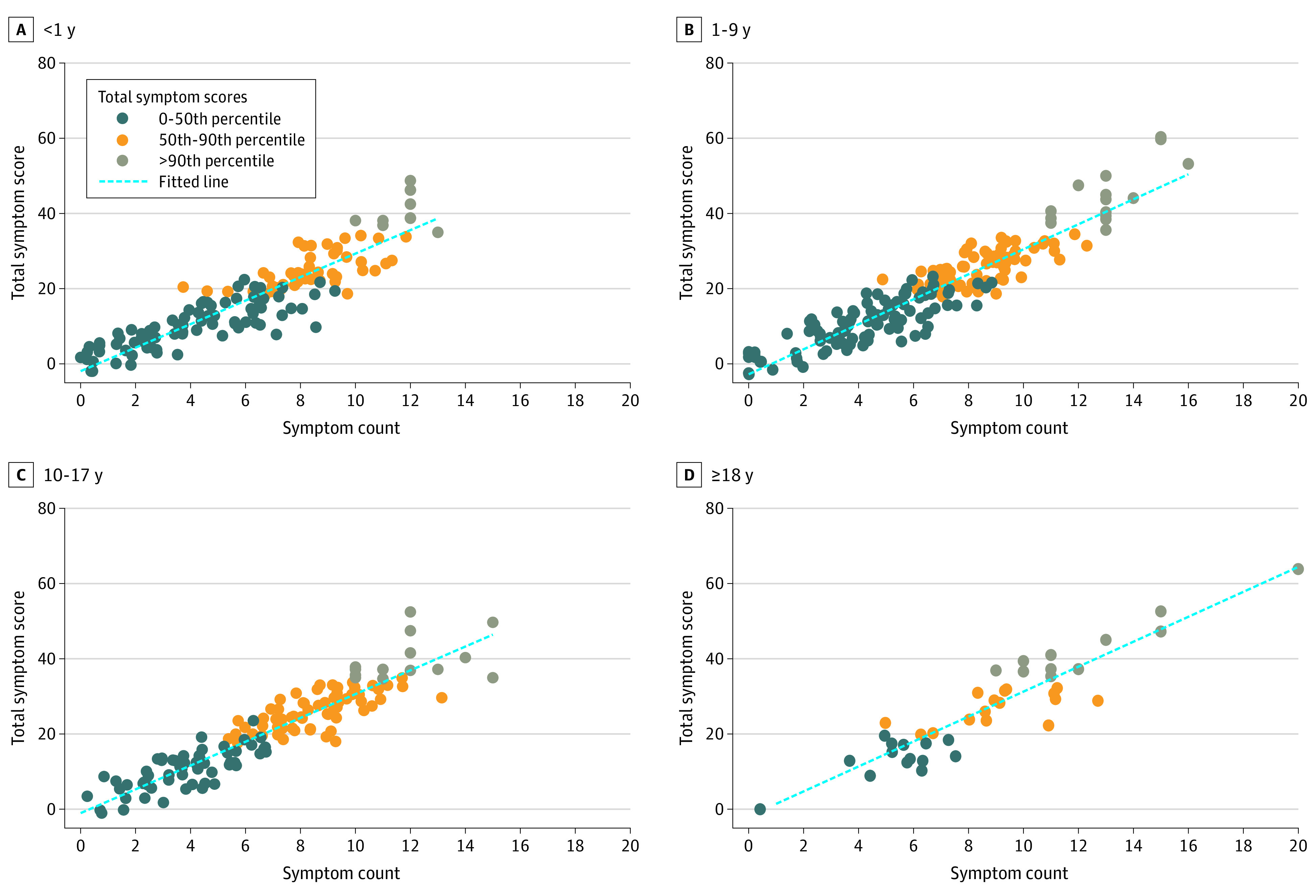
Association of Symptom Counts With Total Symptom Score, Stratified by Age Group

Last, given the association of patient age with symptom counts and scores, we stratified patients into 4 age groups (corresponding closely to defined age stages^19^; 0-11 months, 1-9 years, 10-17 years, and ≥18 years), thereby allowing for potential nonlinear symptom and age associations to be detected, and we plotted a 2-way gradient of patients’ total number of symptoms and total symptom scores, identifying the top 10th percentile of patients regarding total symptom scores (Figure 4). Patients in this most symptomatic group had a median of 12.0 symptoms (IQR, 11.0-13.0), which had a mean (SD) severity and frequency scores of 42.0 (7.1) (indicating ratings just below “moderate” and “sometimes”). This group of patients had been receiving PPC services for a mean (SD) of 279 (557) days, compared with a mean (SD) of 372 (797.5) days for all other patients, but this difference was not statistically significant owing to the wide variation in both groups.

## Discussion

Parent-reported symptom data from a large cohort of 501 patients receiving PPC at 7 different children’s hospitals across the United States reveal that these patients experience a wide variety of symptoms of various frequencies and levels of severity. A total of 73.3% of patients experienced polysymptomatology, consisting of 5 or more symptoms (analogous to the count of medications for the definition of polypharmacy^20^). Markedly elevated total symptom scores among many of the patients suggest substantial symptom-related comorbidity, tending to increase from a mean (SD) total symptom score of 12.3 (9.5) among infants younger than 1 month of age to a mean (SD) score of 24.5 (12.8) among patients 18 years or older.

Five aspects of our findings warrant discussion. First, similar to previous research, we found that parents of children with a life-threatening illness report that many of these children experience severe or frequent symptoms, with pain, fatigue, irritability, and shortness of breath being most common.^7,8,15,21^ Our results differ somewhat from reports of children with progressive conditions, which found that the most prevalent symptoms included behavioral symptoms such as agitation,^22^ feeding problems,^9^ and sleep difficulties,^9^ but these differences may have more to do with differing terminology for the same symptoms (such as *irritability* vs *agitation*) or with different assessments of similar underlying or overlapping constructs (such as *loss of appetite* vs *feeding difficulties*). Our results also differ somewhat from those of a study of children with cancer that found that the most prevalent symptoms included loss of appetite and nausea,^23^ but they are similar to those of a previous study of children with advanced cancer, wherein pain and low energy were the most prevalent symptoms.^7^ These findings suggest that symptom profiles may vary depending on underlying conditions (and their treatments) but that symptoms for a variety of advanced serious illnesses converge on a set of common presentations.

Second, among patients receiving PPC, 1 group experiences very high total symptom scores (Figure 4). Furthermore, the fact that neither the number of symptoms present nor the total symptom score was associated with the duration of time that a patient has been receiving PPC services argues that further improvements in symptom assessment and effective management are needed. Steps in this direction should include the development of practice guidelines, quality improvement efforts, and clinical research.^6^

Third, most of the patients experience polysymptomatology. This was observed for all age groups (except neonates <1 month), among patients who had diagnoses in each of the different (but non–mutually exclusive) CCC categories, and among all patients with 2 or more CCC diagnoses. Polysymptomatology underscores the need for systematic assessment because the symptom that might be evident to the clinician is likely not the only symptom and may not be the most problematic one from the patient or parent perspective.^24^ Once accurate inventory and assessment of all present symptoms have been made, polysymptomatology also raises the likely need to prioritize symptom management interventions, potentially posing tradeoffs whereby the effective management of one symptom may make another symptom worse. Clinical management optimization may require using fewer medications to minimize the risks of complex polypharmacy regimens.^25^ Fewer medications can be achieved in part by selecting medications that ameliorate more than 1 symptom, in part by avoiding medications when nonpharmacologic interventions are effective and in part by deciding which symptoms, lower levels of disruption of function or reduction of quality of life, can simply be tolerated.

Fourth, our observed mean symptom count of 6.7 symptoms is greater than a previous study’s mean of 3.2 symptoms,^9^ most likely because we assessed 20 possible symptoms compared with the 7 possible symptoms in the previous study. When we restricted our symptom count to only 7 symptoms, similar to those assessed in the previous study (pain, difficulty sleeping, vomiting, constipation, shortness of breath, seizures, and drowsiness), our sample’s mean symptom count was 2.9. This finding underscores the importance that how symptoms are assessed (in clinical practice or research studies) is associated with the ensuing data. Furthermore, we observed that patients with different CCC categories were reported to be experiencing approximately the same number of symptoms and total symptom scores. Although the prevalence of different specific symptoms varied across CCC categories (eg, see the lower prevalence of cough among patients with malignant neoplasm CCCs or the lack of energy among infants with neonatal CCCs in Figure 3), if a symptom was present, that symptom’s score was similar across all patients with that symptom (Figure 3).

Fifth, these findings highlight important additional questions and issues to be surmounted. The potential bidirectional association between child symptoms (as reported by parents) and parent mental well-being needs to be better understood. Identification of symptom clusters among pediatric patients may yield insights to guide clinical investigations and management.^26^ Studies of longitudinal symptom and symptom cluster trajectories, and how these are associated with specific treatments, are critical.^27^ Expanding studies to include parents who speak languages other than English and Spanish is important.

### Strengths and Limitations

This study has strengths and limitations. Like patient-reported outcomes data, parent-reported data regarding a child’s symptoms offer a complementary, if not more accurate and superior, view of a child’s experience, compared with clinician-reported data.^5,28^ Both parent and clinician assessment of symptoms in infants and in older patients who are unable to communicate are challenging, especially for symptoms such as low energy or dry mouth, which may not have clear observable manifestations.^29,30^ Patient-reported ratings for a symptom may be associated, broadly speaking, with that patient’s mental state (such as anxiety or despondency).^31,32,33,34,35^ Given that having a child receiving palliative care services also negatively affects the mental state of parents,^36,37,38,39^ a set of complex reciprocal relationships between child and parent are likely encoded into parent-reported data.^40^

## Conclusions

This study suggests that patients receiving PPC experience diverse symptoms and that the most symptomatic patients experience multiple symptoms with high severity and frequency ratings. The assessment and management of polysymptomatology are critical aspects of PPC.

## Notes

Supplement 1. **eTable.** Symptoms Assessed by Age-Range-Specific Symptom Assessment Measures **eFigure.** Total Symptom Score and Number of Days Patients Had Received Pediatric Palliative Care Prior to Baseline Assessment

Supplement 2. **Nonauthor Collaborators.** PPCRN SHARE Project Group
