# Supplementary material for: Polysymptomatology in Pediatric Patients Receiving Palliative Care Based on Parent-Reported Data
Source: JAMA Netw Open. 2021 Aug 5;4(8):e2119730. doi: 10.1001/jamanetworkopen.2021.19730 (PMC8343495; doi:10.1001/jamanetworkopen.2021.19730)
Supplement: Supplement 1. — eTable. Symptoms Assessed by Age-Range-Specific Symptom Assessment Measures eFigure. Total Symptom Score and Number of Days Patients Had Received Pediatric Palliative Care Prior to Baseline Assessment [file jamanetwopen-e2119730-s001.pdf]

Supplementary Online Content

Feudtner C, Nye R, Hill DL, et al; Pediatric Palliative Care Research Network Shared Data and Research (PPCRN SHARE) Project Group. Polysymptomatology in pediatric patients receiving palliative care based on parent-reported data. *JAMA Netw Open.* 2021;4(8):e2119730. doi:10.1001/jamanetworkopen.2021.19730

eTable. Symptoms Assessed by Age-Range-Specific Symptom Assessment Measures
eFigure. Total Symptom Score and Number of Days Patients Had Received Pediatric Palliative Care Prior to Baseline Assessment

This supplementary material has been provided by the authors to give readers additional information about their work.

© 2021 Feudtner C et al. *JAMA Network Open.*

eTable. Symptoms Assessed by Age-Range-Specific Symptom Assessment Measures

	Under 2 years of age			2 years of age and older		
	Presence	Severity	Frequency	Presence	Severity	Frequency
Pain	x	x	x	x	x	x
Lack of energy	x	x	x	x	x	x
Cough	x	x	x	x	x	x
Dry mouth	x	x	x	x	x	x
Drowsy	x	x	x	x	x	x
Difficulty sleeping	x	x	x	x	x	x
Urination	x	x	x	x	x	x
Vomiting	x	x	x	x	x	x
Short of breath	x	x	x	x	x	x
Diarrhea	x	x	x	x	x	x
Sweats	x	x	x	x	x	x
Itching	x	x	x	x	x	x
Difficulty eating/appetite	x	x	x	x	x	x
Difficulty swallowing	x	x	x	x	x	x
Irritable	x	x	x	x	x	x
Constipation	x	x		x	x	
Changes in skin	x	x		x	x	
Mouth sores	x	x		x	x	x
Seizures	x	x	x	x		
Bleeding	x	x	x	x		

Legend:
Note that, in addition to presence, severity, and frequency, symptom “bother” was also assessed for the older children but was not assessed for children under 2 years of age.

© 2021 Feudtner C et al. *JAMA Network Open.*

eFigure. Total Symptom Score and Number of Days Patients Had Received Pediatric Palliative Care Prior to Baseline Assessment

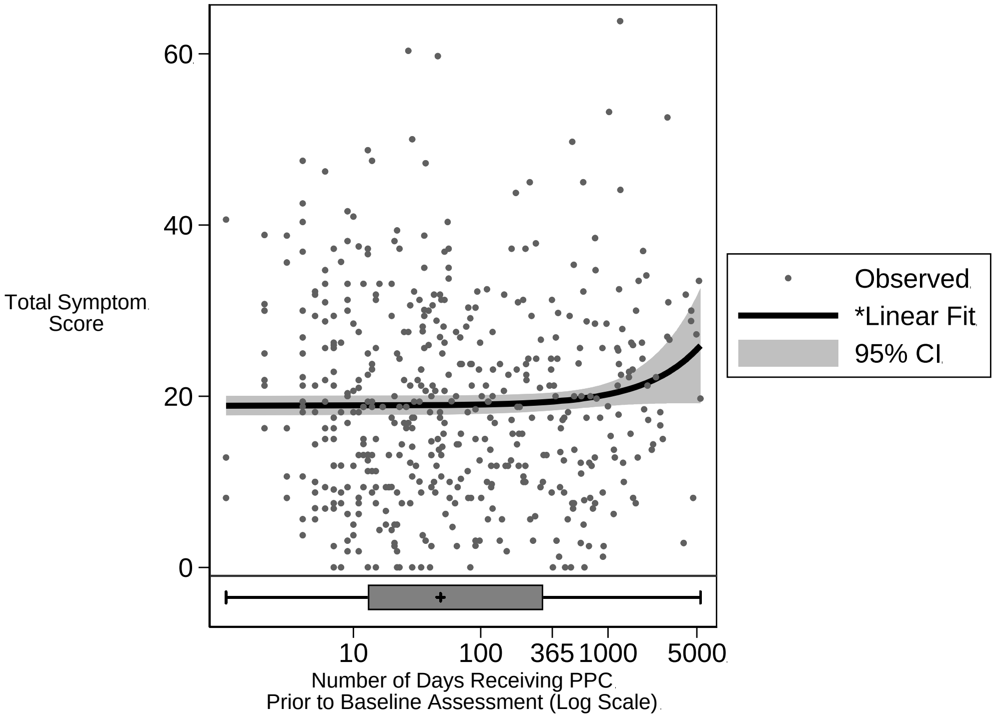

Legend:
Upper portion of figure displays a scatter plot of the number of days receiving pediatric palliative care (**PPC**) prior to the baseline assessment and the total symptom score, along with fitted linear regression line and 95% confidence interval (CI).

*The slope of this line did not differ significantly from zero (P= 0.06), and in a model that adjusted for the age of the patient (which is confounded with time receiving PPC prior to baseline assessment), the relationship remained insignificant (P=0.3).

The lower portion of the figure displays a box-and-whiskers plot of the distribution of the number of days receiving PPC prior to the baseline assessment, with the + symbol indicating the median value of 48.5 days, the left end of the box indicating the 25th percentile value of 13 days, the right end of the box the 75th percentile value of 310 days, and the ends of the thin line indicating the range from 0 to 5329 days.

© 2021 Feudtner C et al. *JAMA Network Open.*
