# Supplementary material for: Polysymptomatology in Pediatric Patients Receiving Palliative Care Based on Parent-Reported Data
Source: JAMA Netw Open. 2021 Aug 5;4(8):e2119730. doi: 10.1001/jamanetworkopen.2021.19730 (PMC8343495; doi:10.1001/jamanetworkopen.2021.19730)
Supplement: Supplement 2. — Nonauthor Collaborators. PPCRN SHARE Project Group [file jamanetwopen-e2119730-s002.pdf]

Supplemental Online Content: Nonauthor Collaborators

*Indicates required information. Only first name, last name, and suffix will appear in PubMed.

*Group Name(s): PPCRN SHARE Project Group							
***First Name and Middle Initial(s)**	***Last Name**	***Suffix (eg, Jr, III)**	Academic Degrees	Institution	Location (city, state/province, country)	Role or Contribution, eg, chair, principal investigator	Group (if more than 1 Group listed in the byline) and/or Subgroup (eg, Steering Committee)
Karen	Crew		BS	Children's Hospital of Philadelphia	Philadelphia, PA, USA	Project coordinator	PPCRN SHARE Project Group
Hannah	Katcoff		MPH	Children's Hospital of Philadelphia	Philadelphia, PA, USA	Database design/mgm	PPCRN SHARE Project Group
Heather	Griffis		PhD	Children's Hospital of Philadelphia	Philadelphia, PA, USA	Database design/mgm	PPCRN SHARE Project Group
Rae	Xiao		PhD	Children's Hospital of Philadelphia	Philadelphia, PA, USA	Statistical analysis	PPCRN SHARE Project Group
Kris	Catrine		MD	Children's Hospitals and Clinics of Minnesot	Minneapolis, MN, USA	Site PI	PPCRN SHARE Project Group
Tatiana	Arevalo-Soriano		BS	Texas Children's Hospital	Houston, TX, USA	Site coordinator	PPCRN SHARE Project Group
Leah	Beight		MPH	DFCI/Children's Hospital Boston	Boston, MA, USA	Site coordinator	PPCRN SHARE Project Group
Madeline	Bilodeau		MPH	DFCI/Children's Hospital Boston	Boston, MA, USA	Site coordinator	PPCRN SHARE Project Group
Jennifer	Chapman		MPH	Children's Hospital of Philadelphia	Philadelphia, PA, USA	Site coordinator	PPCRN SHARE Project Group
Porag	Das		BS	DFCI/Children's Hospital Boston	Boston, MA, USA	Site coordinator	PPCRN SHARE Project Group
Gabrielle	Helton		BA	DFCI/Children's Hospital Boston	Boston, MA, USA	Site coordinator	PPCRN SHARE Project Group
Rachel	Jenkins		MA	Akron Children's Hospital	Akron, OH, USA	Site coordinator	PPCRN SHARE Project Group
Ali	Kolste		MS	Children's Hospitals and Clinics of Minnesot	Minneapolis, MN, USA	Site coordinator	PPCRN SHARE Project Group
Deborah	Maglionico		MS	Akron Children's Hospital	Akron, OH, USA	Site coordinator	PPCRN SHARE Project Group
Isaac	Martinez		BA	Children's of Alabama	Birmingham, AL, USA	Site coordinator	PPCRN SHARE Project Group
Amanda	Mercer		BA	Seattle Children's Hospital	Seattle, WA, USA	Site coordinator	PPCRN SHARE Project Group
Ashley	Morris		BS	Akron Children's Hospital	Akron, OH, USA	Site coordinator	PPCRN SHARE Project Group

Supplemental Online Content: Nonauthor Collaborators

*Indicates required information. Only first name, last name, and suffix will appear in PubMed.

***First Name and Middle Initial(s)**	***Last Name**	***Suffix (eg, Jr, III)**	Academic Degrees	Institution	Location (city, state/province, country)	Role or Contribution, eg, chair, principal investigator	Group (if more than 1 Group listed in the byline) and/or Subgroup (eg, Steering Committee)
Shimei	Nelapati		BS	Texas Children's Hospital	Houston, TX, USA	Site coordinator	PPCRN SHARE Project Group
Rachel	Porth		BA	DFCI/Children's Hospital Boston	Boston, MA, USA	Site coordinator	PPCRN SHARE Project Group
Nicole	Etsekson Sherr		MPH	Seattle Children's Hospital	Seattle, WA, USA	Site coordinator	PPCRN SHARE Project Group
Kelly	Shipman		MS	Seattle Children's Hospital	Seattle, WA, USA	Site coordinator	PPCRN SHARE Project Group
Namrata	Walia		MD	Texas Children's Hospital	Houston, TX, USA	Site coordinator	PPCRN SHARE Project Group
